# Newspaper Jubilee

**Published:** 1871-11

**Authors:** 


### NEWSPAPER JUBILEE.

Very few papers, religious or secular, now published, have been in existence more than a quarter of a century; but the *New York Observer* announces that it will enter upon its fiftieth year in the beginning of 1872. It was established as a religious paper; giving, also, the most important secular news; and it has been one of the ablest, and at the same time, one of the most successful journals in the country.

The publishers announce for the coming year, as a free gift to each of their subscribers, a NEW YEAR-BOOK; containing a vast amount of information in regard to Church and State, and all important business affairs, a real encyclopedia, such as any intelligent person wishes to have always at hand. Specimen copies of the paper and Prospectus of the New Year-Book, sent free to all who will apply. New Subscribers will receive the paper free until January 1st.

WE are sorry that, unavoidably, we have not sufficient space in this issue of THE COMPANION, to give a notice of our Books and Pamphlets. Several other very important articles are, also, crowded out.

